# Cough aerosol in healthy participants: fundamental knowledge to optimize droplet-spread infectious respiratory disease management

**DOI:** 10.1186/1471-2466-12-11

**Published:** 2012-03-21

**Authors:** Gustavo Zayas, Ming C Chiang, Eric Wong, Fred MacDonald, Carlos F Lange, Ambikaipakan Senthilselvan, Malcolm King

**Affiliations:** 1 Mucophysiology Laboratory, Department of Medicine, Faculty of Medicine and Dentistry, University of Alberta, Edmonton, AB, Canada; 2 Department of Medicine, Faculty of Medicine and Dentistry, University of Alberta, Edmonton, AB, Canada; 3 Centre for Lung Health, Northern Lung Function Laboratory, Edmonton General Hospital, Edmonton, AB, Canada; 4 Department of Mechanical Engineering, Faculty of Engineering, University of Alberta, Edmonton, AB, Canada; 5 Department of Public Health Sciences, School of Public Health, University of Alberta, Edmonton, AB, Canada

## Abstract

**Background:**

The Influenza A H1N1 virus can be transmitted via direct, indirect, and airborne route to non-infected subjects when an infected patient coughs, which expels a number of different sized droplets to the surrounding environment as an aerosol. The objective of the current study was to characterize the human cough aerosol pattern with the aim of developing a standard human cough bioaerosol model for Influenza Pandemic control.

**Method:**

45 healthy non-smokers participated in the open bench study by giving their best effort cough. A laser diffraction system was used to obtain accurate, time-dependent, quantitative measurements of the size and number of droplets expelled by the cough aerosol.

**Results:**

Voluntary coughs generated droplets ranging from 0.1 - 900 microns in size. Droplets of less than one-micron size represent 97% of the total number of measured droplets contained in the cough aerosol. Age, sex, weight, height and corporal mass have no statistically significant effect on the aerosol composition in terms of size and number of droplets.

**Conclusions:**

We have developed a standard human cough aerosol model. We have quantitatively characterized the pattern, size, and number of droplets present in the most important mode of person-to-person transmission of IRD: the cough bioaerosol. Small size droplets (< 1 μm) predominated the total number of droplets expelled when coughing. The cough aerosol is the single source of direct, indirect and/or airborne transmission of respiratory infections like the Influenza A H1N1 virus.

**Study design:**

Open bench, Observational, Cough, Aerosol study

## Background

Since the early 1990s the World Health Organization (WHO), along with other governmental and non-governmental agencies, has issued multiple requests to the scientific community. These requests have been for contributions in the development and design of novel approaches, methods, and technologies to optimize management of infectious respiratory diseases (IRD) in anticipation of new and re-emerging transmissible respiratory diseases, such as the Influenza Pandemic and Tuberculosis (TB).

The WHO reported that around one third of the world's population are carriers of *Mycobacterium tuberculosis*, the bacillus that leads to active TB. Annually, nine (9) million new cases of active TB are reported around the world in young and middle aged adults, with about 1.7 million deaths in 2009.

Currently, the Influenza Pandemic Preparedness Plan developed by the WHO considers vaccination as the main support to prevent disease and death from epidemic-prone and pandemic-prone IRD, anti viral are only considered secondary support [1-6]. Most countries around the world have adopted this plan.

Textbooks in Medicine and in other health related areas teach that infectious respiratory diseases such as Tuberculosis and Influenza have a common symptom: cough. Those textbooks also teach that via cough is how these diseases are spread and transmitted to non-infected susceptible individuals. Cough mechanisms are described in those textbooks with emphasis in clinical diagnostic and management of the individual with cough [7-9].

Public Health authorities promote and recommend simple non-pharmacological interventions (NPI), such as hand washing, respiratory hygiene/cough etiquette, facemasks, school closures, and social distancing or isolation to prevent transmission of droplet-spread epidemic-prone diseases. However, researchers like Morse, consider that most of these NPI are based on weak scientific evidence [10]. Moreover, the unexpected outbreak of the severe acute respiratory syndrome caused by a coronavirus (SARS-CoV) [11], together with the outbreak of the highly pathogenic avian influenza H5N1 virus (2005), brought to the forefront the need to find new and more effective IRD transmission control measures [12-16].

IRD are a leading cause of morbidity and mortality in humans. They cause great disruptions in many sectors - economic, educational, recreational, and familial - and can bring worldwide healthcare systems to near collapse. IRD, whether bacterial, mycotic or viral, are transmitted to non-infected persons when an infected individual expels droplets loaded with pathogenic microorganisms during coughing.

There are three components required for the transmission of any respiratory pathogen: a) the transmissor (infected person), b) the surrounding environment, and c) the recipient (non-infected person). Some additional considerations include the concentration of infectious droplets determined by the volume of the space and its ventilation, the length of time of exposure, as well as the status of defense mechanisms of the exposed individual.

Regarding the transmission of IRD, there are many widely accepted facts: a) IRD are transmitted via droplets originating in the respiratory system of an infected individual, b) non-infected individuals could be infected via direct, indirect and/or airborne route, c) infection could occur at a very short distance but also at a very long distance, and d) cough is the most representative source of droplets expelled as aerosol. Consequently, viruses such as Influenza A H1N1 are transmitted when an infected patient expels droplets of different sizes loaded with pathogenic microorganisms, to the surrounding environment as an aerosol during coughing [17-22].

Despite these facts, there is still an ongoing debate to determine whether the transmission of influenza from person to person occurs either primarily through inhaling micron-sized droplets (an airborne route), or through direct or indirect contact with larger sized droplets (physical contact route).

The end result of this debate will have a direct influence on social distancing (i.e. how far apart people should position themselves to prevent infection) and on whether current recommended primary prevention measures and commonly used personal protective equipment are effective barriers to transmission.

What is clear is that the influenza virus requires a mucosa as the entry point to the body. The most vulnerable areas include the mucosa of the eyes, mouth, throat, and the vast surface area of the upper and lower airways and lung tissue. Droplets less than 2.5 microns dry quickly, remain airborne, and reach deep into the lungs when inhaled. On the other hand, large-size droplets are propelled into the environment during coughing and land on nearby surfaces, potentially becoming a source of transmission when standard and/or contact precautions are not followed as recommended.

The unexpected emergence of the H1N1 virus, that triggered two waves of pandemic Influenza A in 2009 [23], confirmed the need to close those important gaps in knowledge regarding the routes of transmission of droplet-spread diseases as well as to optimize NPI.

Coughing is the second most important mechanism, after mucociliary action, in the clearance of respiratory secretions, as well as the most common symptom of many infectious and non-infectious respiratory diseases. In this article we adhere to definition of cough provided by the European Respiratory Society (ERS): *Cough is a three-phase expulsive motor act characterized by an inspiratory effort (inspiratory phase), followed by a forced expiratory effort against a closed glottis (compressive phase) and then by opening of the glottis and rapid expiratory airflow (expulsive phase) *[24].

Therefore, acquiring a deeper insight into airway droplet break up and dispersion during coughing will be invaluable for designing sound evidence-based preventative measures and practices. This will complement and enhance protection to the general public, first responders, and frontline health care workers when caring for and transporting patients to and from healthcare institutions.

Mucus performs an essential role in maintaining a steady and strictly controlled homeostasis in several systems of the body, including the respiratory system. Mucus is a dynamic vehicle with a complex biochemical composition, capable of exerting unique physical properties. Viscosity and elasticity are the physical characteristics considered essential to mucus function, in addition to other important characteristics, such as adhesivity and spinnability.

Two mucus clearance mechanisms exist in the airways: ciliary clearance and cough clearance. Low frequency or low amplitude is closely related to ciliary clearance, while high frequency/high amplitude is more closely related to cough clearance. This bimodal response of the mucus to different frequencies is better explained with a simple and practical analogy: the seat belt. The seat belt responds or functions differently depending on the impulse or tension that is applied. If one pulls it slowly (low frequency), it can be extended easily, but if it is pulled suddenly (high frequency), it holds one tightly to the seat and cannot be extended.

Coughing causes both mucus aerosolization and droplet generation. When the layer of mucus lining the airways interacts with the high-speed airflow of the expulsive phase (up to100 km/h) [25,26] droplets of different sizes are formed and forced up the airway tree, during which droplet collision and coalescing may occur. Hence, droplets coming out of the mouth of a coughing individual will very likely be a mixture of various sizes, generated at different levels of the respiratory systems, and of diverse compositions.

Despite what is known, a large and critical knowledge gap was encountered when no characterization of a cough aerosol model in humans or in animals was found in the literature searched. Other than the general suggestion to "*cover your mouth when coughing*" or to voluntarily quarantine yourself (i.e. "*if you have flu symptoms, please delay your visit*"), we found no evidence-based information on procedures or techniques regarding cough aerosol control at the source, the respiratory system of an infected individual.

Previous attempts by Zayas *et al. *to develop a mammalian cough aerosol model yielded no success (2007, non-published data). This prompted us to strive for the development of a human cough aerosol model, as well as to enhance our knowledge and understanding on the bioaerosol pattern during coughing.

Our main objective was to develop a standard human cough aerosol model to acquire deeper knowledge and understanding of the human bioaerosol pattern to best characterize the number and/or size of droplet production contained in the cough aerosol. To achieve this we required a fast acquisition and high-resolution system, the laser diffraction technique, to capture the cough droplet size and number distribution to overcome the limitation of velocity and evaporation.

Since Duguid [27], (1946) examined the droplet size distribution using direct micrometry on oiled glass slides, droplet size technology has evolved to different instruments, including: *optical droplet counter *(Fairchild 1987; Papineni 1997; Edwards 2004; Schwarz 2010), *aerodynamic droplet sizer *(Yang 2007, Morawska 2009), *scanning mobility droplet sizer *(Yang 2007), *electrical low pressure impactor *(Hersen 2008), *interferometric Mie imaging *(Chao 2009), *droplet image velocimetry *(Chao 2009), and *laser diffraction system *(Edwards 2004). All these previously used measurement techniques have either limited resolution in the submicron range, or have bias due to sampling air stream. Tang *et al. *(2009) have obtained a qualitative real-time characterization of the cough aerosol in human volunteers using an optical technique known as *Schlieren *[27-37].

Our goal is to enhance the knowledge on cough bioaerosol, regarded as the source of direct, indirect and/or airborne respiratory disease transmission, to develop a cough aerosol model in humans, and to set the stage for future studies that will apply novel interventions to reduce the aerosolizability of respiratory secretions as a surrogate of transmissibility.

## Methods

### Study design

This was an observational study in which all the participants, in an open bench format, were encouraged to give their best effort to voluntarily elicit a "real cough" three separate times.

### Participants

A total of 45 healthy non-smokers, male/female volunteers 18 years of age or older, consented to participate. Participants were recruited through advertised leaflets in public areas around a university campus and none of them declared to having asthma, Cystic Fibrosis, or other respiratory conditions. Eligible participants were excluded if they had received expectorants, mucolytics or natural products for respiratory conditions during the previous 30 days, or had developed a flu-like illness immediately before the study.

### Study site

The study was carried out at the Mucophysiology Laboratory, 173 Heritage Medical Research Centre, University of Alberta, Edmonton, Alberta, Canada.

Environmental conditions at the study site were similar to the indoor conditions found in a hospital reception site with respect to room temperature, humidity and atmospheric pressure.

The University of Alberta Hospital Medical Ethics Committee and the Office of Environmental Health and Safety of the University of Alberta approved the study protocol. Informed consent was obtained from all subjects.

### Study day

The study procedures were explained in detail to all participants by the investigator. Once they had understood the study requirements, all participants were asked to sign an informed consent.

### Aerosol pattern analysis

For accurate, time-dependent droplet size distribution analysis, a laser diffraction system (Spraytec, Malvern, UK) was used. The laser diffraction system has 60 size bins with the capability of measuring the concentration of droplet sizes from 0.1 micron (μm) to 900 μm every 0.4 millisecond.

The Spraytec He-Ne (Helium-Neon) laser diffractometer is composed of transmitter and receiver modules. Expelled respiratory aerosols pass through a cylindrical measurement zone with a volume of 7.85 cm^3 ^through a path of 100 mm length and 10 mm diameter. The path length is estimated as the distance through the spray plume that the laser beam travels. As the droplets pass through the laser measurement volume zone, laser light from the transmitter is scattered by the respiratory aerosol producing light diffraction patterns, which are measured by optical detectors on the receiver modules. The light signals are then converted into electrical signals to process a droplet size distribution, under the assumption that each droplet is a perfect sphere. The angle at which a droplet diffracts light is inversely proportional to its size.

The He-Ne laser diffractometer was set to measure the droplet concentration of a single cough event crossing the measurement zone every 0.4 milliseconds (2.5 GHz) during a manually triggered time of 1.5 seconds.

### Statistical analysis

The data were expressed as mean ± standard deviation (SD) unless otherwise stated. Fulfillment of normal log distribution was asserted by the Shapiro-Francia test [38]. A paired Student *T*-test was used for simple comparison. For multiple comparisons between groups a two-way ANOVA test was used. A value of p < 0.05 was considered significant.

## Research procedures

### Cough aerosol

**Figure 1:**
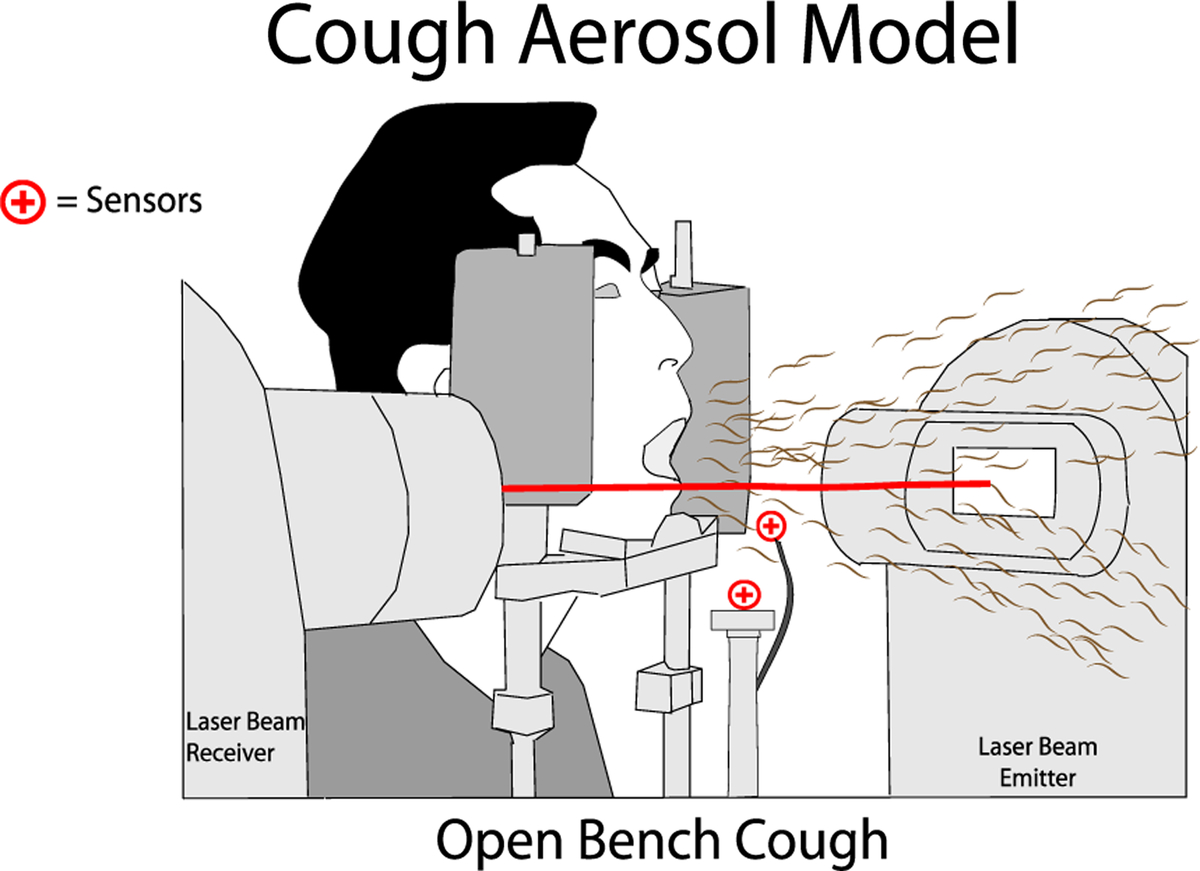
**Laser and sensor arrangement for Cough Aerosol detection**.

Participants were asked to place their head in a modified device similar to the head brace used by optometrists and were asked to perform a "real cough". To assess the cough maneuver a laser beam was directed from left to right in front of and parallel to the participant's face, at approximately 17 cm distance from their mouth to the centre of the beam's measurement zone. Since there was no precedents regarding the use of a laser beam in an open bench format to assess cough aerosol, the 17 cm distance was a decision made by the researchers. This decision was based mainly on the grounds of safety: to avoid contact of the laser beam with the eye or face of the participant. They were positioned to allow the cough airflow jet to cross the beam without any interference to the flow of the aerosol as seen below (Figure 1). Opposite to the participants an open fume hood removed airborne particles from the environment. We did not measure evaporation rate. Deposition was not a factor in the open bench design.

## Results

During the period of study, March - May 2010, we detected inside the testing site an average in atmospheric pressure of 91.8 ± 1.1 KPa, in relative humidity of 19.0 ± 3.9% RH, and in temperature of 22.7 ± 2.0°C. The rate of air exchange in the study site (six to nine air exchanges per hour) was lower than in a hospital emergency room.

Aerosol droplets expelled during a single cough event were assessed in 26 male and 19 female participants self-identified as non-smokers, with the exception of one male who declared he was a long-term (30+ years) ex-smoker.

Every participant was encouraged to voluntarily elicit a "real cough" three times. If during the performance researchers considered that the participant did not make an adequate effort the participant was ask to repeat the maneuver until getting an acceptable effort.

In addition to the acceptable cough efforts, we consistently selected three parameters provided by the laser beam machine: valid points, skip values and total mass per maneuver. From these parameters we decided and selected which maneuver was the best. The design implemented in our study was based similarly to when performing a spirometry test: three efforts and select the best effort made. This is a procedure in lung mechanics very well establish and accepted worldwide.

Full and detailed results were obtained in less than five minutes after coughing. The average and standard deviation of weight, height, Body Mass Index (BMI) and age of all participants is shown in Table 1.

**Table 1:** Anthropometric data of 45 participants

n = 45	Weight (kg)	Height (cm)	BMI (kg/m^2)	Age
Average ± SD	66.4 ± 12.9	167.9 ± 9.1	23.5 ± 4.0	34.3 ± 15.2

Table 2 shows the coefficient correlation of weight, height and corporal mass against number and diameter of droplets, including the value of r and p value.

**Table 2:** Coefficient Correlation data weight, height and BMI

n = 45	Weight	Height	BMI
**Average Diameter**			

r	-0.14	-0.05	-0.13

p-value	0.36	0.74	0.39

**Number**			

r	0.24	0.20	0.13

p-value	0.11	0.19	0.39

All participants (n = 45)

The average mass of the cough aerosol that crossed the measurement volume zone, derived from the aerosol volume concentration, was 2.2 milligrams (mg) for the 45 participants. The cough expulsion phase lasted an average 700 milliseconds.

The large number of different sized droplets, generated by the best-effort cough and detected in the 60 bins by the laser diffractometer, were normalized and expressed as the average rate of *number of droplets per cubic centimeter per second*. These averages were grouped into six (6) categories: a) < 0.5 μm, b) 0.5 to 1 μm, c) > 1.0 to 2.5 μm, d) > 2.5 to 10 μm, e) > 10 to 100 μm and f) > 100 μm; then tabulated by age and sex (Table 3) and summarized in Table 4.

**Table 3:** Average rate of cough droplet size by sex and age

N = 45	Age ≤ 30		30 > Age ≤ 50		Age > 50	
**Sex**	**Male**	**Female**	**Male**	**Female**	**Male**	**Female**

N < 0.5 μm	1.33E+07	5.32E+06	5.27E+06	1.75E+07	4.53E+07	1.65E+07

0.5 μm < N < 1.0 μm	1.89E+05	2.46E+05	1.83E+05	4.06E+05	2.79E+05	2.25E+05

1.0 μm < N < 2.5 μm	3.45E+04	1.12E+04	1.81E+04	4.22E+04	2.69E+04	3.95E+04

2.5 μm < N < 10.0 μm	4.01E+04	1.24E+04	1.78E+04	4.75E+04	4.41E+04	4.51E+04

10 μm < N < 100 μm	1.86E+03	4.95E+02	7.71E+02	3.30E+03	3.46E+03	2.71E+03

N > 100 μm	0.00E+00	0.00E+00	0.00E+00	0.00E+00	0.00E+00	0.00E+00

Unit: # droplets/cc/second

**Table 4:** Average rate of cough droplet size by age group (outlier removed)

N = 44	Age ≤ 30	30 > Age ≤ 50	Age > 50 Exclude outlier
N < 0.5 μm	1.02E+07	1.05E+07	2.89E+07

0.5 μm < N < 1.0 μm	2.15E+05	2.78E+05	2.48E+05

1.0 μm < N < 2.5 μm	2.58E+04	2.84E+04	3.41E+04

2.5 μm < N < 10.0 μm	2.96E+04	3.05E+04	4.47E+04

10 μm < N < 100 μm	1.34E+03	1.85E+03	3.03E+03

N > 100 μm	0	0	0

Unit: # droplets/cc/second

Data obtained from the cough maneuvers in 44 participants (outlier removed) showed a large variability in the number of droplets in all droplet size categories and the standard deviation was very large.

Acquired data indicates that 97% of droplets, expelled in one second during coughing, are smaller than 1 μm, 2.7% of droplets are between 1 - 10 μm. Hence, our data indicates that 99% of the total of droplets expelled, when a healthy non-smoker coughs, are droplets smaller than 10 μm, i.e. *inhalable *droplets.

Participants were categorized as low emitters/high emitters if their data was one standard deviation below/higher than the average of the population. Seven (7) participants were identified as low emitters, and ten (10) as high emitters. One ex-smoker (30 + years) was identified as a high emitter. Another high emitter (> 50 years old) was beyond two standard deviations greater than the average and considered an outlier.

The outlier is a very fit athlete who practices high intensity sports. The mass of the cough aerosol of the athlete outlier amounts to 32 mg, compared to the average of 2.2 mg. When removing the outlier from the rest of participants, the tabulated data showed very similar data to the other two age groups as seen in Table 4. Data presented from now on will be with the outlier removed.

**Figure 2:**
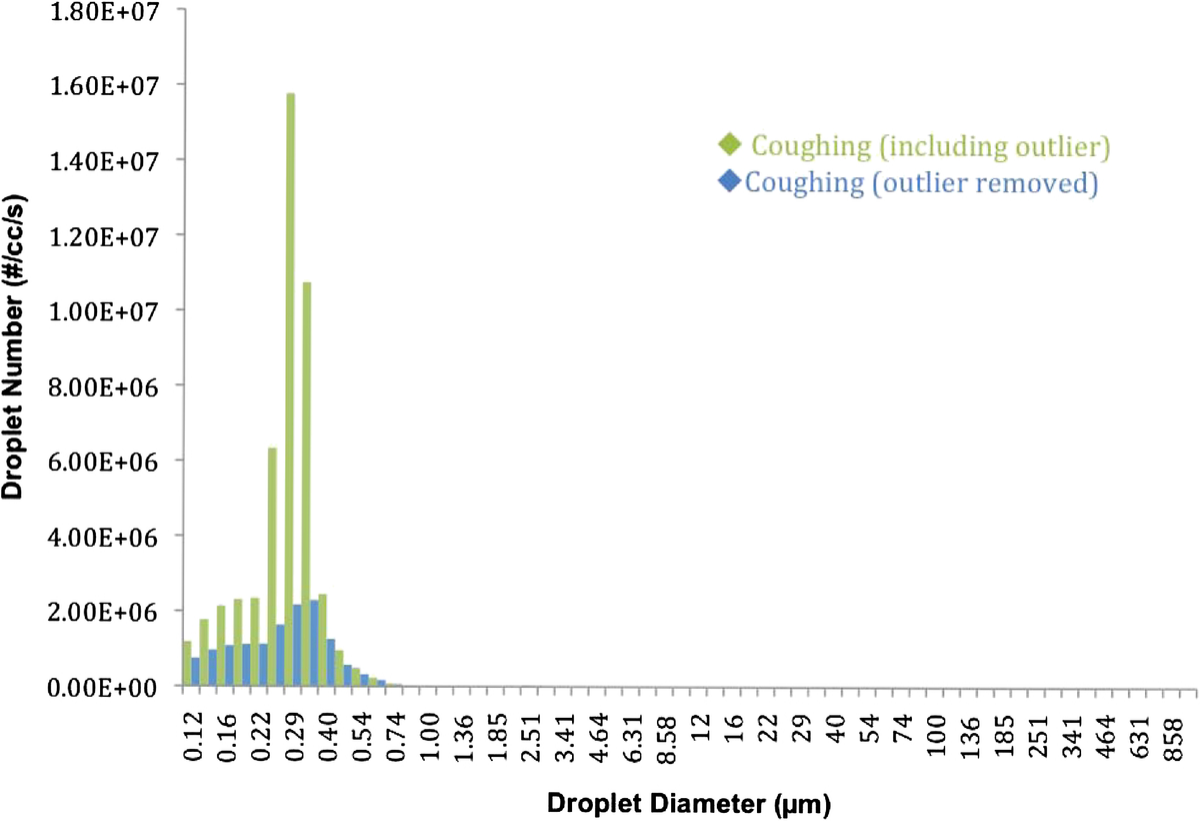
**Full spectrum characterization of cough aerosol number versus droplets diameter per second**.

Accounting for more than 97% of the total number of droplets per cough, droplets smaller than 1 micron were the most numerous of all. Cough produces a larger number of droplets per second in every droplet size category; see Figure 2 below.

Due to the enormous amount of droplets of smaller diameter, columns representing the larger droplet diameters did not show up in the graph. While it seems that no droplets were detected in the higher ranges, there were in fact a few droplets (e.g. 63 μm = 42 droplets, 86 μm = 14 droplets, 100 μm = 7 droplets, 293 μm = 1 droplet).

**Figure 3:**
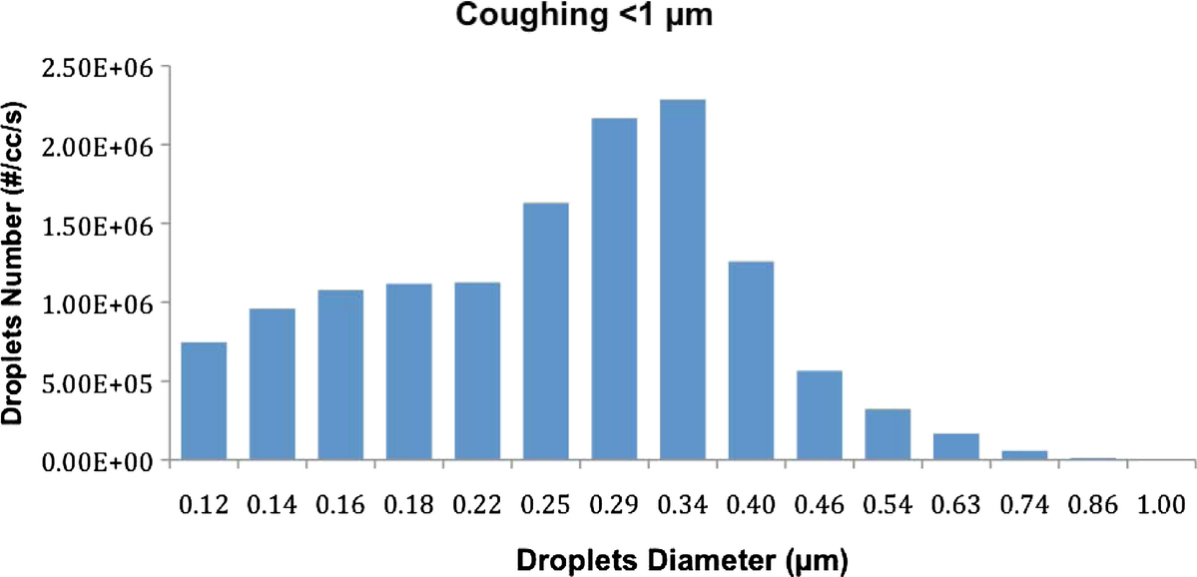
**Quantities of measured droplets in size category < 1 μm per second**.

Figure 3 represents 97% of all droplets measured. In Figures 3.1 - 3.4 (found in Additional file 1) we show the entire spectrum of droplets per size in all bins, expelled as aerosol when coughing.

Detailed characterizations for the remaining droplet size categories are included as appendices. Detection of emitted droplets during cough lasts about 700 milliseconds and has a tri-modal distribution.

We performed a two-way analysis of variance (ANOVA) on the data per each category of droplet size per age and per sex, with and without the outlier. Table 5 shows the ANOVA tests for the < 0.5 μm droplet size.

**Table 5:** Two-way ANOVA results after removing the "outlier"

N < 0.5 μm					
**Source**	**Sum Sq**.	**d.f**.	**Mean Sq**.	**F**	**Prob > F**

AGE	2.53E+15	2	1.27E+15	2.36	0.11

GENDER	5.66E+14	1	5.66E+14	1.05	0.31

AGE*GENDER	2.04E+15	2	1.02E+15	1.90	0.16

Error	2.04E+16	38	5.37E+14		

Total	2.47E+16	43			

The remaining ANOVA tests on the other droplet size categories are included as appendixes.

## Discussion

The emphasis of this study was on the development and refinement of a cough aerosol model in healthy human volunteers, detected and verified with a laser diffraction system.

Major findings in this study include: a) the respiratory system generates droplets of many different sizes during coughing; b) droplets smaller than ten-microns account for up to 99% of the total number of droplets that are expelled as a bioaerosol during coughing; c) due to its size distribution and amount, the cough bioaerosol has the potential to contribute directly, indirectly and/or through airborne route to the transmission of respiratory infections, including Influenza A caused by the H1N1 virus; d) age, sex, weight, height and corporal mass have no effect on the size and number of emitted droplets; e) our approach has the potential to identify high emitters and/or outliers; f) these results create a foundation for the development of a standardized human cough aerosol model; g) the acquired data creates a foundation for the development of tools in airway hygiene for secretion management, as well as in prevention of droplet-spread illnesses.

During the preparatory phase of this study, our research group was concerned that healthy non-smokers would find it difficult to voluntarily perform a reproducible "*near*-*real-cough aerosol*". We considered requesting participants to undergo respiratory challenges such as inhaling hypertonic saline to induce augmented airway secretion and/or capsaicin to elicit a "real cough". The concern was due to technical facts: the thickness of the airway mucus layer in healthy non-smokers is 5 to 10 microns [39,40].

The International Organization for Standardization (ISO) has stated "*the laser diffraction technique has evolved such that it is now a dominant method for determination of droplet size distribution". (ISO 13320:2009 (E) 2009) *[41]. While testing and tuning the laser diffraction system we found that our initial concerns were inaccurate: Healthy non-smokers are excellent models to characterize cough aerosol droplets. Thus, we avoided using any challenging intervention that could alter the physical properties of the airway mucus layer.

The first limitation of this study was that the laser diffraction system, according to the manufacturer, was not intended to assess aerosolized mucus from the airways when coughing. This created an uncertainty as to whether we could capture any droplets at all. However, thanks to some unique technical expertise in our group, our lab was able to use the machine for our intended purposes, if slightly limited.

Other limitations were related to the performance of the laser diffraction system with a fluid that has optical properties different than water. Berge and Pearce report that the Refraction Index of Air is 1:0 and of Water is: 1.3, and Reid reports that the refractive index of mucus is very similar to that of pure water [42,43].

Properties of mucus are different in health than during disease. Mucus in healthy individuals is more opaque than water. In diseased state, mucus opaqueness could be more pronounced further affecting optical properties. During bacterial infection there is formation of pus or mucus could become bloody, altering most physical and optical properties.

However, during acute viral respiratory infections such as flu-like diseases, respiratory secretions are watery and clear. Therefore our assumption is valid for influenza, SARS-CoV, and avian influenza, which remain our priority. To assess cough aerosol in diseases like Tuberculosis, Cystic Fibrosis or others caused by bacteria, we may need to determine the optical properties of diseased mucus first.

Any droplets travelling in the periphery of the plume and outside of the measurement zone are unaccounted for. Our experiment was designed to capture a representative section of the cough plume crossing the path length and the measurement zone, including droplets from the lateral periphery that cross the measurement section. We estimate that we captured a sample of 15% of cough droplets that are representative of the cough plume but we have no definitive way or method to accurately determine this yet.

There is a confounder we have not deal with yet, and is the contribution of saliva to the number and size of droplets detected. This is a topic for the next trials.

Linear correlation indicates a very weak association between height, weight and BMI with the size and number of cough droplets expelled. These findings lend further support to the concept that cough droplet diameter/number distribution is mainly determined by the physical properties of the layer of mucus, such as elasticity, cohesiveness. A mucus layer with low elasticity and poor cohesiveness due to infections (i.e. the watery mucus layer during a flu-like disease) or mucus exposed to respiratory agents that disrupt bondings tends to break apart with more ease. Consequently, this will form a larger number of droplets of different size.

A mucus layer with strengthened elasticity and high cohesiveness will be more resistant to break, and will form less number of droplets and/or produce fewer droplets of a larger size. This is a concept we described in previous publications [44,45].

In healthy non-smoker individuals there is an optimum range among their mucus physical properties that allow it to behave in a balanced manner even at different frequencies. This enabled us to reach a milestone: enhance our understanding of the cough aerosol role in droplet-spread, pandemic-prone IRD.

Nevertheless, several factors observed in our design suggested that the "best effort" cough requires improvements. Specifically, the distance from the mouth to the laser beam and the position of the face, were identified as factors to further assess and improve in order to minimize the variability of the acquired data.

The open bench design was selected since we were interested in characterizing the cough bioaerosol in an indoor environment that could simulate and explain what would happen in a real-life emergency room, triage site, school, home, or any enclosed location where people gather. This approach would facilitate assessing the characterization of the bioaerosol coming out the respiratory system and dispersed into the surrounding environment.

Indoor conditions in the study site were maintained at similar room temperature, humidity, and atmospheric pressure as in the reception site of a hospital, with the exception of a lower rate of air exchange. Hence, the open bench format will not require a translation into real-life situations, unlike enclosed formats. It took our group a great deal of effort to overcome the limitation of the system and extract the number of droplets in the cough bioaerosol, since the laser diffraction system does not explicitly provide it. We have not encountered any information of any research team assessing cough droplets using an open bench format.

Researchers from various disciplines around the globe have dedicated a large number of studies to the investigation of cough aerosol droplets, using a variety of study designs, as well as multiple quantitative and qualitative methods and techniques. During our brief literature review, we found several key differences between those studies and our methods: they all used closed systems of various designs to assess the respiratory droplets; the majority of them used equipment with much lower resolution, limited range of sizes and biased droplet collection to characterize the size and number of droplets; and almost all of them used equipment with much lower data acquisition speeds than the one used in our study [27-37]. Without a doubt, these differences played a critical role in explaining why our data differs from the majority of data reported in the literature.

Furthermore, our research group considers that it is fundamental to reach a consensus in defining the components of the cough aerosol. Current terminology linked to cough aerosol and IRD transmission/dispersion (e.g. particles), are similar to terms used in air quality studies, where pollutants or "particles" are mostly composed of solid materials and gases generated during combustion process.

In this article our research group consistently uses the term droplets instead of particles to define the components of the cough aerosol, since water content dominates the composition of the airway mucus (~ 95%), with solid content filling out the remaining percentage. A consensus will enhance our effectiveness in IRD management and protection by reducing confounding terms. A consensus is also needed to clarify how the influenza virus is transmitted.

The high proportion of droplets smaller than one micron (97%), expelled as aerosol in a single cough, are susceptible to rapid evaporation when released to an environment with different humidity and temperature than inside the airways. This supports the probability that an airborne route of transmission could be a dominant force in the transmission of droplet-spread IRD. Interestingly, a group of researchers led by Palesi [46,47], have published several studies indicating that, using a small mammalian model, a viral infection was transmitted to animals in different cages connected only by a tube with no direct contact involved. Airborne droplets, emitted by the infected group of animals, are the most likely mode of transmission in such a model. Hence, the contribution of droplets smaller than one micron in viral transmission merits further investigation.

Data from this study allow us to not only characterize the cough aerosol, but also to identify outstanding emitters. 10 individuals were categorized as high emitters of cough droplets. One of the high emitters was beyond two standard deviations greater than the average number of droplets expelled when coughing and was considered as an outlier, and the other nine only one standard deviation apart from the mean.

Data from our participants indicate that age, sex, weight, height or corporal mass have no statistically significant effect on the aerosol composition in terms of size and number of droplets, as confirmed by linear correlation assessment (Table 2) and ANOVA tests (Table 5). Results of the ANOVA test including all participants showed a tendency that did not reach a statistically significant difference in the following all droplet size categories. Excluding the outlier removed the tendency towards a difference that is statistically significant.

These results coincide with previous findings by Zayas (MSc Thesis, 1989) that viscoelastic properties, determined by rheology, from tracheal mucus do not differ in young and old healthy male/female adults who are non-smokers, including those mature non-smokers with pulmonary restrictive diseases [48].

The high emitter outlier was identified as a very fit athlete who practices high intensity sports. Such physical activities would very likely have a positive effect on lung mechanics, hence, allowing for a better lung capacity. However, it is tempting to speculate that if such a person happens to develop influenza they could become a "super-spreader" due to the high number of droplets expelled when coughing. Figure 2 illustrates the enormous difference between this outlier and the other participants. The same figure also highlights that droplets smaller than one micron (< 1 μm) clearly dominate over the rest of the droplets sizes.

Regarding the tri-modal size distribution, the third size mode at ~251 μm (between 215 - 464 μm size) is very small in our study. This third mode is of such small magnitude when compared with the other two modes that it is only evident in annexed Figure 3.4. Johnson in a recent publication, using a different methodology and technique, also reported a third mode peaking at around 200 μm [49]. We are preparing another article where we will discuss in more detail similarities and differences of our method and results from other teams of researchers working on the same topic. Our team of researchers is fully convinced that only a multi and trans-disciplinary collaboration will provide the optimal strategy to best manage, reduce infection, disease, and death due to IRD in rich and poor countries alike.

Another high emitter of cough droplets is a self-identified long-term (+ 30 years) ex-smoker. Previous studies found that viscoelastic properties of tracheal mucus from subjects exposed to tobacco smoke determined by rheological analysis are different (p < 0.05) with respect to the non-smoker population [50-53]. The remaining eight high emitters identified themselves as non-smokers. There was no investigation to verify if they have had any type of voluntary or involuntary exposure to tobacco smoke, or to other airborne insults.

Pharmacological and non-pharmacological factors are capable of disrupting the optimum balance of the physical properties found in mucus. Compounds that break-up and lyse the cross-linking binding sites at different levels of the mucin glycoprotein gel network can subsequently affect the natural balance of viscosity and elasticity of the airway mucus. Thus, transforming it in a less cohesive or more watery fluid, facilitating airway mucus droplet formation. This is an unexplored area that needs to be addressed.

A literature search yielded no scientific or empiric information regarding the effect that natural and/or non-natural compounds may exert on aerosolization of mucus during respiratory condition treatment. This is an area that also merits detailed research due to the wide and common use of these compounds and in light of recent epidemic-prone and pandemic-prone droplet-spread IRD. Therefore, it is critical to determine and grade the effect of both pharmacological and/or non-pharmacological factors on airway mucus aerosolization.

Seven participants were identified as Low emitters. Initially, we interpreted this result as a technical issue: that during the study these participants for some reason directed the cough airflow jet in a direction that prevented it to cross the measurement zone of the laser beam. The other possibility we are contemplating is that these participants may have inherent physical properties in their airway mucus that makes it more resistant to break up into droplets when coughing. There may be other explanations and we are preparing to explore these in future studies.

Based on the results obtained in this study we are confident that we have achieved a strategic and critical step towards optimizing management of droplet-spread epidemic-prone infectious diseases in the form of a detailed real-time characterization of the cough aerosol regarding the size and, more importantly, the number of droplets expelled when coughing.

The ERS definition of cough does not mention anything about droplets formed and expelled as a direct result of the interaction of the high-speed airflow with the layer of airway mucus during the expulsive phase. A consensus panel report on "*managing cough as a defense mechanism and as a symptom*" [54], endorsed by the American College of Chest Physician, the American Thoracic Society, and the Canadian Thoracic Society, made a detailed, highly clinical description of coughing. However, it does not discuss anything regarding cough aerosol droplets as the vehicle of transmission of IRD.

The fact that several of the most prestigious international professional organizations dealing with lung health and/or respiratory diseases, including the Council of Canadian Academies, are not discussing droplet formation and expulsion during coughing as a critical factor in IRD transmission and dispersion indicates that there is a knowledge gap and lack of consensus that require immediate attention [55-59].

This study provides both scientific support as well as encouragement to design evidence-based preventative measures and alternatives in existing technologies to optimize public health practices and personal protection barriers in bioaerosol control to prevent the spread of IRD. The human cough aerosol model could serve as the foundation for the development of an in-vivo, innovative and robust bioaerosol assessment tool. This tool could be quite useful during an IRD outbreak as a point of care diagnostic test for screening, detecting, and monitoring, individuals with an acute respiratory infectious medical condition. Our method yield results in less than five minutes, hence would reduce time inconveniences in scenarios where large amounts of individuals continuously gather; such as emergency entrances in hospitals, airports, bus/train stations, etc. By using an evidence-based preventative screening method, staff working in these scenarios will feel reassured that they have reliable protection.

This technology will complement and enhance protection to first responders and frontline health care workers caring for, and transporting, patients to and from health care institutions. Furthermore, it will also protect the general public during IRD outbreak transmitted by droplets via direct, indirect and airborne route.

Despite the fact that Canada has one of the most advanced healthcare systems in the world, Canadians' health remains at risk from droplet-spread IRD. The success in handling the SARS crisis was questioned, and the lesson to be taken from this experience is that Canada has room to improve in its ability to manage IRD outbreaks, not unlike most countries worldwide [60].

Our research group is aware that our findings clearly differ from other studies, but we also are aware that our findings still need further evaluation before confirmation of our data and findings. For the human cough aerosol model to achieve its full potential, a much larger sample of participants is required; hence ongoing recruitment of subjects is needed for the foreseeable future to confirm its value.

In addition, our research group at the Mucophysiology Laboratory, University of Alberta has been developing a novel non-vaccine strategy, *mucomodulation *[44,45], that has shown the potential to slow or stop the spread of IRD, potentially saving lives as well as reducing the burden on resources for healthcare systems in rich and poor countries alike.

Currently the mucomodulation strategy has evolved into an ongoing comprehensive program known as ***The Edmonton Platform***, and will serve as a complement to the WHO Pandemic Preparedness Plan. This program will be further detailed in a forthcoming article, however, it is conceived as the launching ground for methods and tools of innovative technologies, products, interventions, and strategies suitable to integrate into a country's health system, policies and strategies for IRD outbreak protection.

Data acquired in this study allowed us to achieve our main objective by establishing the fundamental basis of a standard human cough aerosol model. This would enable us all to acquire better knowledge and understanding of the human bioaerosol pattern by best characterizing the number and/or size of droplets production contained in the cough aerosol that might open new avenues in epidemic-prone and pandemic-prone IRD outbreaks preparedness.

## Conclusions

We have further characterized quantitatively the pattern, size, and number of droplets of the most important mode of person-to-person transmission of IRD: the cough bioaerosol.

The results from this study strengthen the concept that cough aerosol contributes to direct, indirect and/or airborne transmission of respiratory infections like the Influenza A H1N1 virus. This knowledge may contribute to limit or eliminate the debate around the main route of transmission of respiratory infections of known and, more importantly, of unknown infectious respiratory pathogens.

The optimal control of droplets contained in the aerosol expelled while coughing could be best achieved by applying an integral approach rather than individual approach. Therefore, we strongly recommend the implementation of an integral strategy such as the Edmonton Platform, which has a potential to become the gold standard in droplet-spread IRD studies, and a Canadian contribution to the world for droplet-spread epidemic-prone, pandemic-prone infectious respiratory disease control.

In later studies we plan to assess the characterization of the infectious bioaerosol coming from the external environment towards the respiratory system of a non-infected individual, and the effectiveness of novel non-pharmaceutical procedures. The voluntary cough practiced by healthy participants in an open bench format could permit us to determine in the near future the amount and type of droplets expelled by an individual infected with Influenza A H1N1 virus or with TB visiting a health care facility.

## Competing interests

The authors declare that they have no competing interests.

## Authors' contributions

JGZ and MK developed the concept of cough aerosol, designed the study, interpreted the data and drafted the manuscript. EW and FM performed clinical assessment of participants, contributed to develop the basis for the human cough aerosol model and with clinical interpretation and review of the manuscript. CL contributed to strengthen the methodology of the study and determine the best data acquisition device and revision of the manuscript. AS contributed with statistical analysis of data and with critical revision and interpretation of the manuscript. MCC made key contributions by making the laser device to operate according to the study design, with acquisition of data, and by developing the software to optimize data acquisition and the database. All authors read and approved the final manuscript.

## Pre-publication history

The pre-publication history for this paper can be accessed here:

http://www.biomedcentral.com/1471-2466/12/11/prepub

## Supplementary Material

Additional file 1 **Appendix 1: Figures portraying the remaining categories of the size in microns and quantities of open bench cough droplets in one second**. Appendix 2: ANOVA tests of the remaining categories of the size in microns and quantities of open bench cough droplets in one second. Click here for file
