# Supplementary material for: Cough aerosol in healthy participants: fundamental knowledge to optimize droplet-spread infectious respiratory disease management
Source: BMC Pulm Med. 2012 Mar 21;12:11. doi: 10.1186/1471-2466-12-11 (PMC3331822; doi:10.1186/1471-2466-12-11)
Supplement: Additional file 1 — Appendix 1: Figures portraying the remaining categories of the size in microns and quantities of open bench cough droplets in one second. Appendix 2: ANOVA tests of the remaining categories of the size in microns and quantities of open bench cough droplets in one second. [file 1471-2466-12-11-S1.DOC]

List of Appendixes

Appendix 1: Graphs portraying the remaining categories of the size in microns and quantities of open bench cough droplets in one second.

Appendix 2: ANOVA tests of the remaining categories of the size in microns and quantities of open bench cough droplets in one second.

APPENDIX 1

Graph 2.2: Droplet quantities that are between 1<x≤2.5 µm in size per second


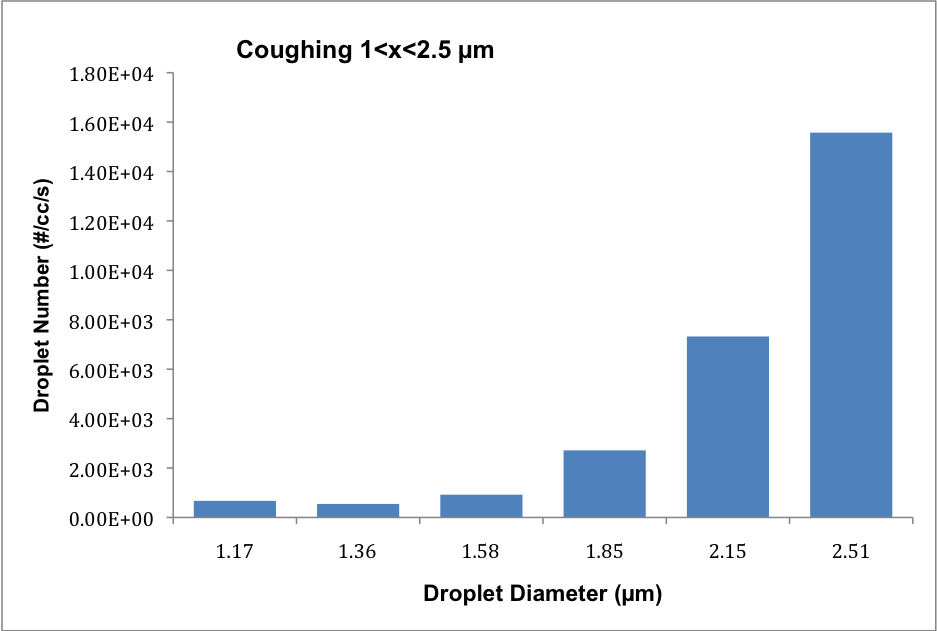


Graph 2.3: Droplet quantities that are between 2.5<x≤10 µm in size per second


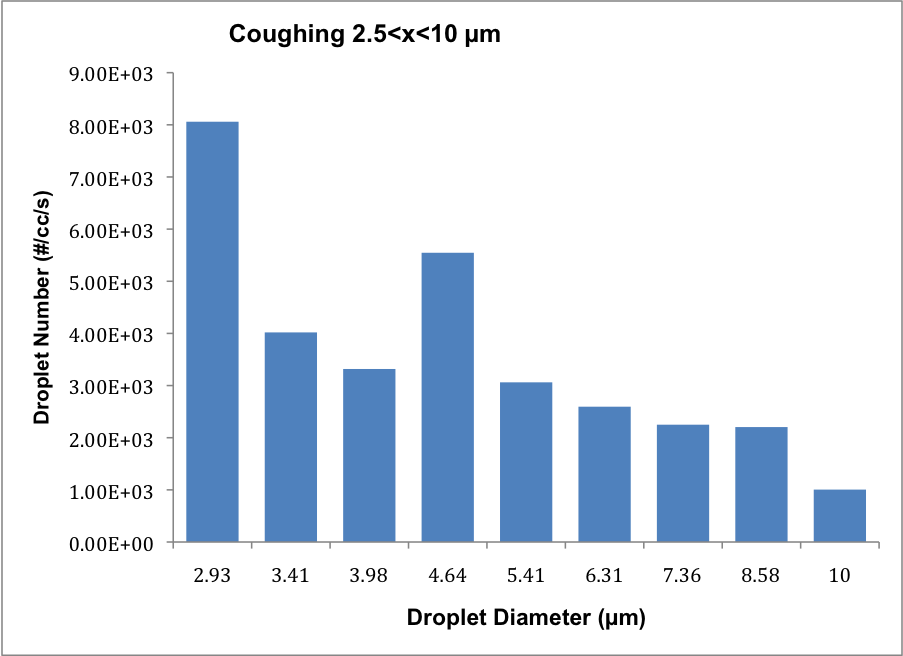


Graph 2.4: Droplet quantities that are between 10<x≤100 µm in size per second


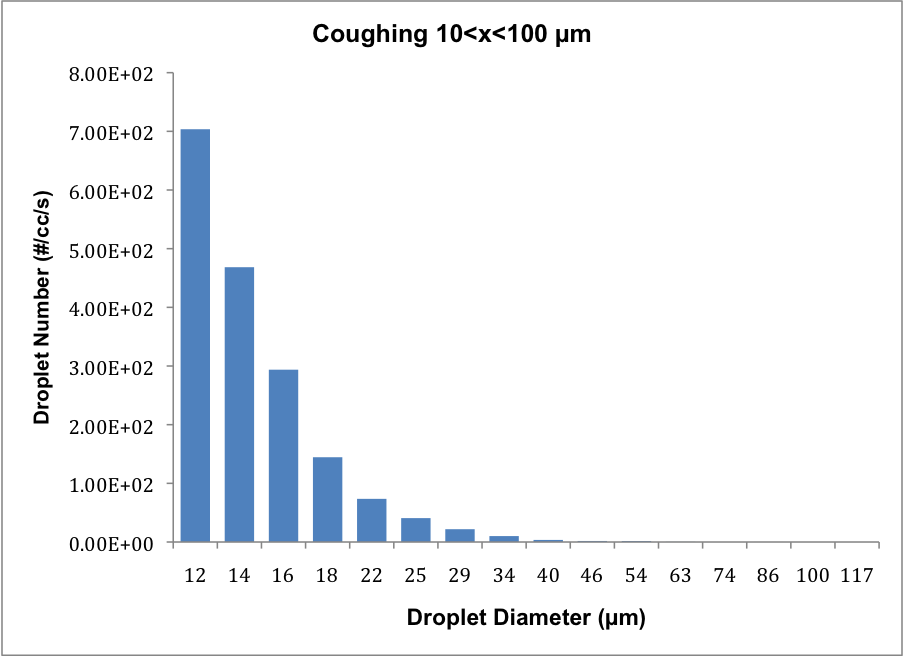


Graph 2.5: Full spectrum characterization of cough-droplets quantities of all sizes in one second (0.1 µm < X< 890 µm)


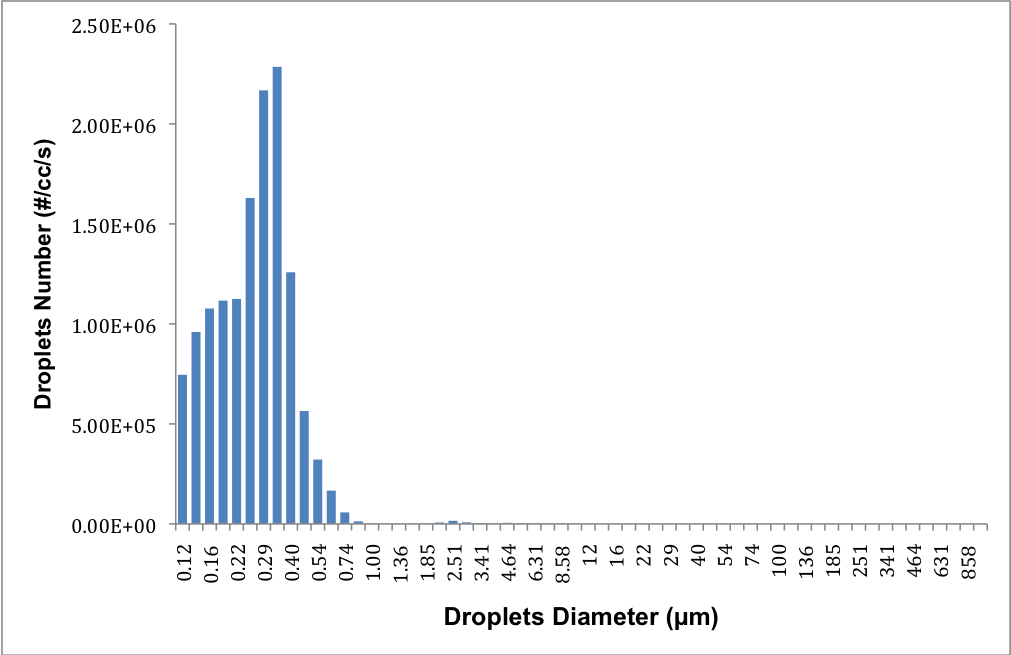


Appendix 2

Table 4.2: Two-way ANOVA results after removing the “outlier”

| **0.5 µm <N< 1.0 µm** | | | | | |
| --- | --- | --- | --- | --- | --- |
| Source | Sum Sq. | d.f. | Mean Sq. | F | Prob>F |
| AGE | 4.97E+10 | 2 | 2.49E+10 | 0.20 | 0.82 |
| GENDER | 4.85E+10 | 1 | 4.85E+10 | 0.39 | 0.53 |
| AGE*GENDER | 1.02E+11 | 2 | 5.10E+10 | 0.42 | 0.66 |
| Error | 4.67E+12 | 38 | 1.23E+11 |  |  |
| Total | 4.90E+12 | 43 |  |  |  |

Table 4.3: Two-way ANOVA results after removing the “outlier”

| **1.0 µm <N< 2.5 µm** | | | | | |
| --- | --- | --- | --- | --- | --- |
| Source | Sum Sq. | d.f. | Mean Sq. | F | Prob>F |
| AGE | 7.70E+08 | 2 | 3.85E+08 | 0.31 | 0.74 |
| GENDER | 1.72E+08 | 1 | 1.72E+08 | 0.14 | 0.71 |
| AGE*GENDER | 5.20E+09 | 2 | 2.60E+09 | 2.09 | 0.14 |
| Error | 4.73E+10 | 38 | 1.24E+09 |  |  |
| Total | 5.29E+10 | 43 |  |  |  |

Table 4.4: Two-way ANOVA results after removing the “outlier”

| **2.5 µm <N< 10 µm** | | | | | |
| --- | --- | --- | --- | --- | --- |
| Source | Sum Sq. | d.f. | Mean Sq. | F | Prob>F |
| AGE | 1.82E+09 | 2 | 9.12E+08 | 0.55 | 0.58 |
| GENDER | 7.12E+06 | 1 | 7.12E+06 | 0.00 | 0.95 |
| AGE*GENDER | 7.05E+09 | 2 | 3.53E+09 | 2.13 | 0.13 |
| Error | 6.30E+10 | 38 | 1.66E+09 |  |  |
| Total | 7.16E+10 | 43 |  |  |  |
